# The impact of recency and adequacy of historical information on sepsis predictions using machine learning

**DOI:** 10.1038/s41598-021-00220-x

**Published:** 2021-10-21

**Authors:** Manaf Zargoush, Alireza Sameh, Mahdi Javadi, Siyavash Shabani, Somayeh Ghazalbash, Dan Perri

**Affiliations:** 1 Health Policy and Management Area, DeGroote School of Business, McMaster University, Hamilton, ON Canada; 2 Department of Industrial Engineering, Amirkabir University of Technology, Tehran, Iran; 3 Department of Decision Sciences, HEC Montréal, Montréal, QC Canada; 4 Department of Biomedical Engineering, Amirkabir University of Technology, Tehran, Iran; 5 Department of Medicine, Faculty of Health Sciences, Department of Critical Care, and Chief Medical Information Officer, McMaster University and Staff Intensivist, St. Joseph’s Healthcare Hamilton, Hamilton, ON Canada

**Keywords:** Diseases, Health care, Medical research

## Abstract

Sepsis is a major public and global health concern. Every hour of delay in detecting sepsis significantly increases the risk of death, highlighting the importance of accurately predicting sepsis in a timely manner. A growing body of literature has examined developing new or improving the existing machine learning (ML) approaches for timely and accurate predictions of sepsis. This study contributes to this literature by providing clear insights regarding the role of the recency and adequacy of historical information in predicting sepsis using ML. To this end, we implemented a deep learning model using a bidirectional long short-term memory (BiLSTM) algorithm and compared it with six other ML algorithms based on numerous combinations of the prediction horizons (to capture information recency) and observation windows (to capture information adequacy) using different measures of predictive performance. Our results indicated that the BiLSTM algorithm outperforms all other ML algorithms and provides a great separability of the predicted risk of sepsis among septic versus non-septic patients. Moreover, decreasing the prediction horizon (in favor of information recency) always boosts the predictive performance; however, the impact of expanding the observation window (in favor of information adequacy) depends on the prediction horizon and the purpose of prediction. More specifically, when the prediction is responsive to the positive label (i.e., Sepsis), increasing historical data improves the predictive performance when the prediction horizon is short-moderate.

## Introduction

Sepsis is a serious medical condition caused by the body’s disrupted response to infection, leading to organ failure, cognitive impairment, long-term functional disability, and even death^1–3^. Because of its significant impact on worldwide morbidity and mortality, it is considered a major public and global health concern^2,4–6^. Each year, sepsis affects more than 30 million people worldwide, leading to about 6 million deaths^7^. It creates an enormous financial burden for healthcare systems, making it among the costliest diseases^8,9^. The estimated cost of hospitalization for treating sepsis in the US alone is $24 billion/year, accounting for nearly 5% of all hospital costs^2,6^.

A convincing body of literature has documented the significant benefits of the early provision of sepsis care in reducing in-hospital mortality, readmission, and length of stay^10–13^. Every hour of delay in detecting sepsis increases the risk of death by 4–8%^14,15^, highlighting the importance of accurately predicting sepsis promptly. However, this is challenging because of the complex nature of the disease, owing to the variety of clinical indications, the sources of infection, and the body’s response to sepsis^16,17^. Considering the widespread electronic health records (EHR) data and the strong computational capabilities available recently, two types of data-driven automated approaches have been used more frequently to predict sepsis and identify patients at-risk. The approach, which is more commonly used in current clinical practices, includes disease severity scoring systems, such as the sequential organ failure assessment (SOFA), systemic inflammatory response syndrome (SIRS), acute physiology and chronic health evaluation (APACHE), simplified acute physiology score (SAPS), and modified early warning score (MEWS), which employ EHR to develop sepsis risk scores^17,18^.

The second approach includes predictive analytics methods, such as machine learning (ML) algorithms. Studies have reported a poorer performance of the traditional risk scoring systems, particularly because of their high alert fatigue and lower efficiency in clinical settings^11^. This is because the rule-based scoring systems are specially designed to identify the risk of sepsis rather than detecting its presence^18^. In contrast, ML approaches employ more complex computational formalisms to optimally utilize the available features in the data and provide sepsis predictions with the lowest cross-validated errors.

A growing body of research in medical informatics and clinical decision support systems is focused on developing new or improving existing ML approaches for timely and accurate predictions of sepsis. However, the literature does not provide clear insights into the role of the *recency* and *adequacy* of historical information in predicting sepsis using ML. We fill this gap by extensively examining the simultaneous impact of these two factors on timely predictions of sepsis. To do this, we introduce the notions of *prediction horizon* and *observation window* to capture information recency and adequacy, respectively. We compare the cross-validated predictive performance of our proposed deep learning (DL) algorithm—namely, bidirectional long short-term memory (BiLSTM)—with six other ML algorithms using several predictive performance measures and under numerous combinations of the prediction horizons and observation windows.

Several recent studies have focused on predicting various sepsis stages using ML algorithms in different clinical settings, reporting a wide performance range for various predictive measures. In the interest of space, we summarize this literature in Table S1 in Appendix A, and we compare our results with this literature in the Discussion section.

## Methods

### Data source, predictors, and outcome variable

**Figure 1:**
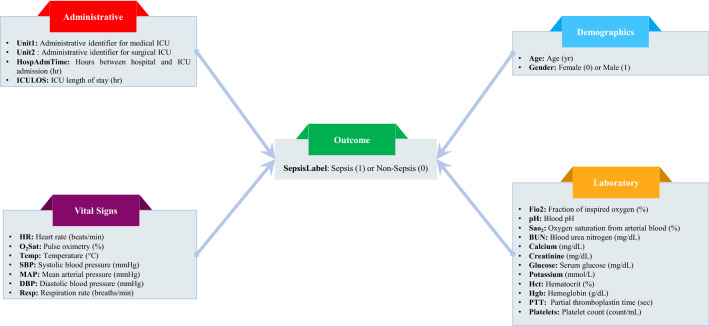
Summary of variables used in the study.

The dataset used in this study, which has been published and publicly available through the 2019 PhysioNet computing challenge^19^, includes EHR data from 40,336 intensive care unit (ICU) patients collected from two hospital systems (Beth Israel Deaconess Medical Center and Emory University Hospital). The approvals have been obtained from the Institutional Review Boards of Emory University, protocol 33069^20^. The full details regarding the dataset are provided in Reyna et al.^21^ and Goldberger et al^22^. All methods were performed in accordance with the relevant guidelines and regulations. This dataset includes a wide range of variables, including two demographic variables, four administrative variables, and 34 clinical variables (8 vital signs and 26 laboratory measurements) recorded hourly, leading to 1,552,210 data records. The sepsis diagnosis was performed based on the Sepsis-3 clinical criteria that were recently developed^2,23,24^. In these data, 2932 patients were labeled septic, indicating a data imbalance. We removed 15 features that had at least 90% missing values and used the remaining variables summarized in Fig. 1.

### Data preparation

In this study, we designed observation windows of different sizes, denoted by $w:w\ge 1$. An observation window of size $w$ includes the longitudinal features backward from time $t$ (most recent section of the window) to time $t-w+1$. Let ${\mathcal{X}}_{t}=\left\{{x}_{1}^{t}, {x}_{2}^{t}, \dots ,{x}_{p}^{t}\right\}$ denote the set of features at time $t$, where ${x}_{i}^{t}$ is the $i$th feature in the feature set and $p$ is the total number of features. Therefore, a window of size $w$ contains all the features in ${\mathcal{X}}_{t}, {\mathcal{X}}_{t-1}$,…,${\mathcal{X}}_{t-w+1}$, representing the amount of historical data used for predictions (a proxy for data adequacy). Associated with each window, we also considered a prediction horizon (a proxy for data recency), denoted by $h$, which is calculated from $t$. It is used to relabel the outcome variable according to the actual timing of sepsis in the data. Accordingly, for each window of size $w$, we define the outcome variable ${y}_{h}^{w}$ to label the sepsis occurrence within $h$ hours from $t$ in the following way:

$${y}_{h}^{w}=\left\{\begin{array}{ll}1, &{\text{if}} \, {\text{sepsis}} \, {\text{has}} \, {\text{occurred}} \, {\text{within}} \, h \, {\text{hours}} \, {\text{from}} \, t\\ 0,& {\text{otherwise}}\end{array}\right.$$

**Figure 2:**
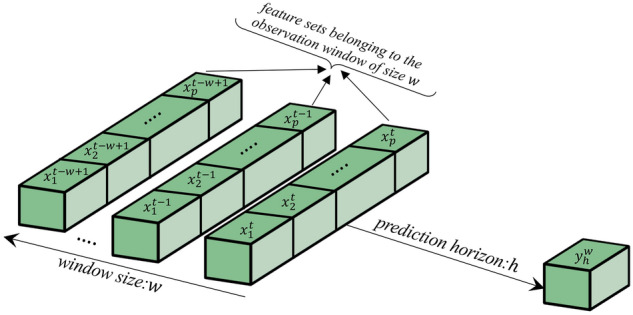
Data organization using observation windows, prediction horizons, and feature set.

Figure 2 depicts all the concepts discussed above. In this study, we conducted predictive analytics using numerous ML algorithms under various combinations of $h$ and $w$ and reorganized the dataset accordingly.

### Handling missing data

Missing values were imputed using the Multiple Imputations by Chained Equation (MICE) algorithm in Python’s Autoimpute package^25^. The algorithm consists of sequential regression analyses (considering the variable type) to generate multiple predictions for each missing value accounting for the relationship between missing value and other variables in the data. It is particularly useful for large imputation procedures; moreover, it is flexible with variable types, and yields robust imputations for sequential data^25,26^. For missing data imputation, we used a modified version of MICE (pseudocode available in Appendix C-III), which avoids using future information to impute missing data in the past. This procedure preserves both causality and real-time application of the algorithm^27^.

### ML algorithms

For an extensive evaluation of the ML predictive performance, we used seven ML algorithms under the same data structure/preparation, model training, and assessment procedures. These ML algorithms include logistic regression (LR), classification and regression trees (CART), extreme gradient boosting (XGB), naïve Bayes (NB), linear discriminant analysis (LDA), AdaBoost (ADA), and BiLSTM. All computations and analyses were conducted using Google Colab, executed on Google’s cloud servers. In the interest of space, we briefly explain the BiLSTM algorithm. We assessed the performance of all examined ML algorithms comprehensively over a wide range of prediction horizons ($h=0, 1, 3, 6, 8, 10, 14, 18, 26$ h) and observation window sizes ($w=1, 2, 4, 6$), leading to 288 full ML analytics.

### BiLSTM algorithm

**Figure 3:**
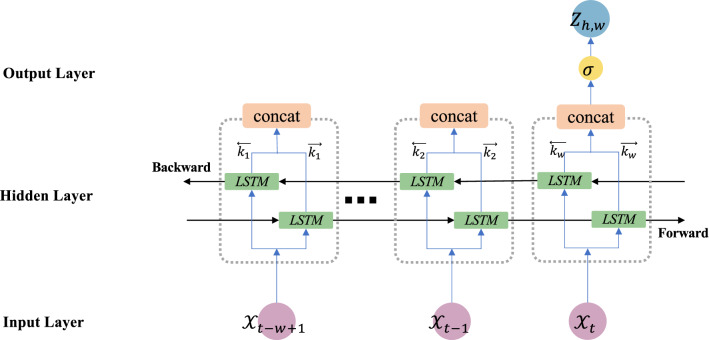
General BiLSTM structure for prediction horizon $h$ using observation window of size $w$.

The key strength of the BiLSTM algorithm is its ability to process sequential data in two directions (forward and backward), where the output layer is connected to two hidden long short-term memory (LSTM) layers. It was introduced as a revised version of the LSTM algorithm^28^, and optimally utilizes historical information (i.e., retaining vs. discarding) using a forget gate^29^. For its bidirectional procedure, BiLSTM considers historical information from both the past and future. For our study, we designed the BiLSTM algorithm as a $w$-step process, as illustrated in Fig. 3. Accordingly, the output sequence of the forward layer, denoted by $\overrightarrow{{k}_{w}}$, is calculated iteratively from $s=1$ to $s=w$, and the output sequence of the backward layer, denoted by $\overleftarrow{{k}_{w}}$, is calculated in the reverse order from $s=w$ to $s=1$. The outputs can then be characterized as ${Z}_{h,w}=g\left(\overrightarrow{{k}_{w}}, \overleftarrow{{k}_{w}}\right)$, where the $g$ function merges the forward and backward sequences.

The bidirectional recurrent neural networks (bidirectional RNN) are not causal models when the prediction task includes multiple outcomes, such as speech recognition or word predictions^30^. In this case, an output can be predicted in the context of future information, hence a violation of causality. This usually occurs in the case of “many-to-many” architecture, when a variable can be both a predictive feature (i.e., input) and output depending on the stage of the prediction. This leads to utilizing future information for predicting outcomes in the past. In the case of the “many-to-one” structure (our case), where the outcome variable in one time is not used as a predictive feature in another time, the above data leakage from future information for predicting the past outcome does not occur^31^. More specifically, we do not use the future knowledge of features or sepsis to make predictions about the past, hence no violation of causality.

### Model training and hyperparameter tuning

For training and assessment of all ML algorithms, we split the dataset into the train (80%), validation (10%), and test (10%) sets using patient-wise splitting criteria to avoid any data leaking and overfitting. We followed this procedure (as opposed to record-wise splitting) because every patient in the data had multiple records. To optimally tune the hyperparameters of the ML algorithms, we used the grid search algorithm, with various optimizers in Python’s Keras library, on the validation data^32^.

### Handling data imbalance

To handle data imbalance, we implemented an oversampling technique employing Python’s SMOTE package to create equal proportions of the smaller class (i.e., Sepsis) and the bigger class (i.e., Non-Sepsis). To avoid any data leaking during this step, we performed data splitting for model training and assessments prior to the oversampling procedure and resampled only the train and validation sets to maintain the natural structure of the data when assessing the predictive performance with the test set.

### Predictive performance measurement and variable importance

For the robust evaluation of the performance of the ML algorithms, we used several measures of predictive performance, including (a) sensitivity (SEN) to evaluate the effectiveness of the classifier to identify the positive label (i.e., Sepsis), (b) specificity (SPE) to evaluate the effectiveness of the classifier to identify negative label (i.e., Non-Sepsis), (c) area under the receiver operating characteristic curve (AUC) to evaluate the tradeoff between true-positive and false-positive rates, and (d) accuracy (ACC) to evaluate the overall prediction accuracy of the classifier. However, because of the importance of predicting the positive label, we were more interested in SEN and AUC. To examine the importance of features, the permutation importance technique^33^ was implemented by randomly permuting the features and examining the resulting impact on the above four predictive performance measures under various combinations of $h$ and $w$.

### Descriptive analysis

Descriptive statistics were reported using general measures of frequency and central tendency.

Our the codes (in Python scripts) for reproducing the results as well as the dataset are publicly available on GitHub (https://github.com/bi-Lstm-for-sepsis-prediction/bi-Lstm-for-sepsis-prediction.git). The password for accessing these items is *Sepsis2021*. All analyses were conducted using Google Colab, executed on Google’s cloud servers.

## Results

### Descriptive results

A descriptive summary of the data is available in the appendix (Table S1 in Appendix B). Accordingly, a total of 1,552,210 observations were included from patients aged between 14 and 100 years. The mean age of the sample was 62 years, with a standard deviation of 16.4 years. Most patients were male (55.9%) and admitted almost equally to surgical and medical ICUs.

### BiLSTM model

Table S2 in Appendix C summarizes the results of hyperparameter tuning for the BiLSTM algorithm, which was conducted by minimizing a loss function (set to binary cross-entropy) using early stopping to prevent overfitting.

**Figure 4:**
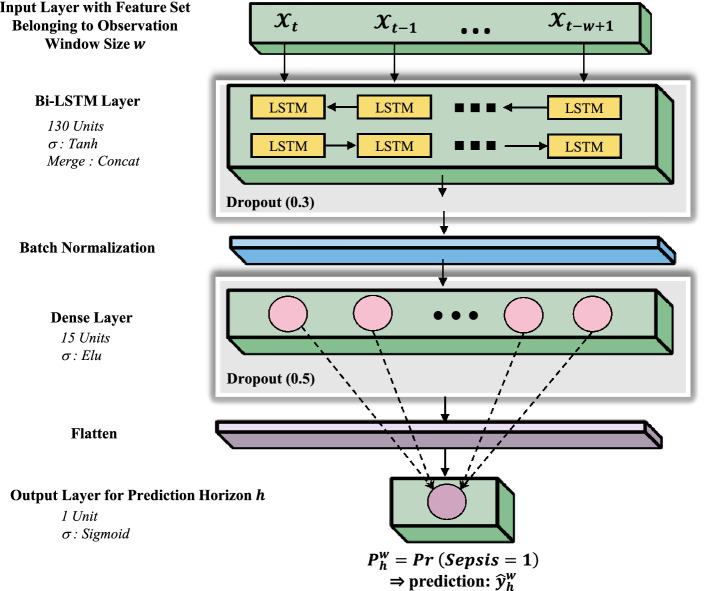
BiLSTM architecture used in this study with prediction horizon $h$ and observation window $w$.

Figure 4 illustrates the BiLSTM architecture used in our study. Accordingly, the BiLSTM layer in our model has two LSTM layers working in two opposite directions (each of 130 neurons with 25 timesteps with the Tanh activation function). The first dense layer has 15 neurons with an Elu activation function, followed by another dropout layer with a dropout rate of 0.5, and the second dense layer has one neuron to provide the predicted probability of sepsis (using the Sigmoid activation function). The dense layers include fully connected neural networks with batch normalization and flatten to further improve the model’s performance.

### Comparing ML algorithms

**Figure 5:**
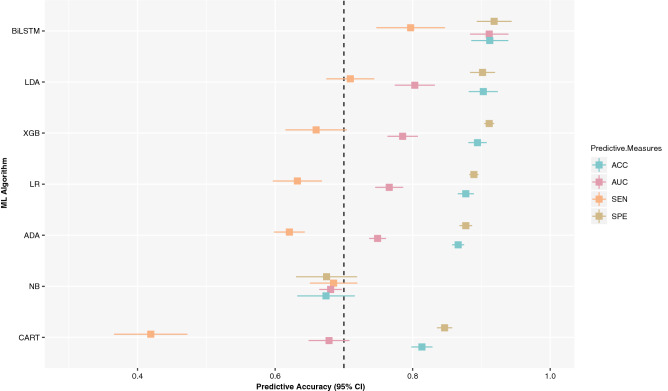
Overall performance of ML algorithms.

First, we assessed the overall predictive performance (averaged over all ranges of $h$ and $w$) of the ML algorithms examined in this study. Figure 5 illustrates the results, which indicate that while most ML algorithms perform equally well based on the ACC and SPE criteria, there is often a performance gap regarding AUC and SEN, which better reflects the predictive performance regarding the positive label (i.e., Sepsis). Accordingly, BiLSTM outperforms all other algorithms (average SEN = 0.80: 95% confidence interval [CI] 0.75–0.85; average AUC = 0.91: 95% CI 0.88–0.94; average SPE = 0.92: 95% CI 0.89–0.94; and average ACC = 0.91: 95% CI 0.89–0.94).

### BiLSTM performance under various prediction horizons ($h$) and observation windows ($w$)

**Figure 6:**
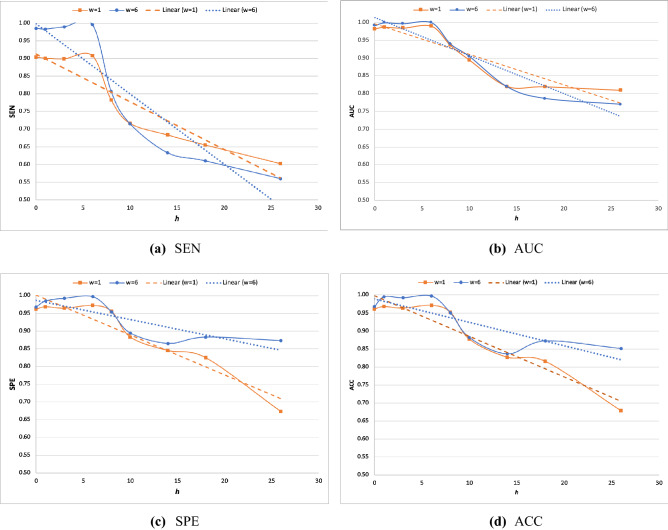
BiLSTM predictive performance.

For the second analysis, we assessed the predictive performance of BiLSTM on a wide range of prediction horizons ($h$). Figure 6 compares the results under the smallest window size ($w=1$) and the biggest one ($w=6$). The results indicate that (i) decreasing the prediction horizon (in favor of information recency) boosts the predictive performance consistently (irrespective of the predictive performance measures or the window size), providing clear evidence for the importance of information recency in improving predictive performance. However, the impact of expanding the observation window (in favor of information adequacy) depends on the prediction horizon and the purpose of prediction. Accordingly, (ii) for SEN and AUC, increasing historical data can boost the predictive performance only when the prediction horizon is short-moderate; otherwise, this leads to reduced predictive performance. In contrast, (iii) for SPE and ACC, increasing historical data always boosts the predictive performance.

Among the results delineated above, result (ii) is particularly remarkable. It states that beyond a certain prediction horizon threshold (e.g., $h\ge 10$ h for SEN or $h\ge 15$ h for AUC), increasing historical information will decrease the predictive performance. This can be better understood by noting that in our modeling, the historical information $w$ is added from the older side (see Fig. 2). For example, while $w=1$ and $w=2$ have ${\mathcal{X}}_{t}$ in common, the added information in $w=2$ compared with $w=1$ is ${\mathcal{X}}_{t-1}$, or $w=3$ contains ${\mathcal{X}}_{t-2}$ in addition to what is included in $w=2$, and so on. This result indicates that when the prediction horizon is long, adding historical information deteriorates the predictive performance regarding the positive label. For example, when $h=1$, changing $w=1$ to $w=2$ means adding data that are 2 h away from the prediction point, whereas the same change under $h=20$ implies adding data that are 21 h away from the prediction point. Our results ascertain that while the additional information under $h=1$ increases the predictive performance regarding Sepsis, the same change under $h=20$ decreases the predictive performance.

### Predicting the risk of sepsis using BiLSTM

**Figure 7:**
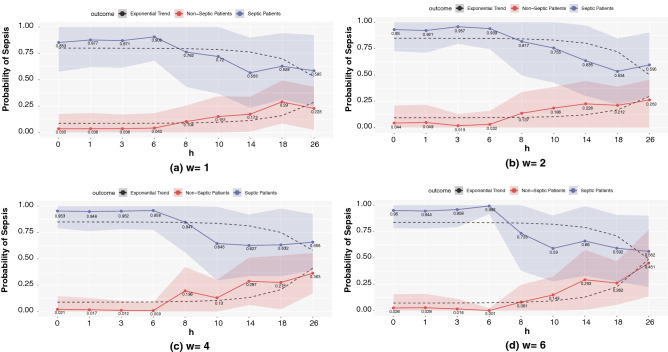
Predicted risk of sepsis among septic vs. non-septic patients.

Our third set of analyses assessed the predicted risk of sepsis for a wide range of prediction horizons under different window sizes. To this end, we applied BiLSTM to a random set of 100 patients and calculated the average probability of sepsis among truly septic and non-septic patients in the set. Figure 7 illustrates the results, which indicate, first, that while there is a great separability of the predicted sepsis risk among septic versus non-septic patients for all window sizes, the separability decreases with prediction horizon $h$, reconfirming the recency phenomenon. Second, and more interestingly, for shorter prediction horizons, the separability is larger when the window size is large (e.g., note the superior separability of 0.95 [for septic patients] vs. 0.026 [for non-septic patients] under $h=0$ and $w=6$ compared with 0.85 vs. 0.035 under $h=0$ and $w=1$), indicating the positive impact of adding historical information (i.e., increasing $w$) on separability when $h$ is small-moderate. The results are the opposite for large prediction horizons (e.g., note the mediocre separability of 0.56 vs. 0.45 under $h=26$ and $w=6$ compared with 0.59 vs. 0.23 under $h=26$ and $w=1$). Indeed, these results are in line with result (ii) above, indicating that adding historical information is useful only when the prediction horizon is short-moderate; otherwise (i.e., when the prediction horizon is long), it reduces the predictive performance.

### Feature importance

**Figure 8:**
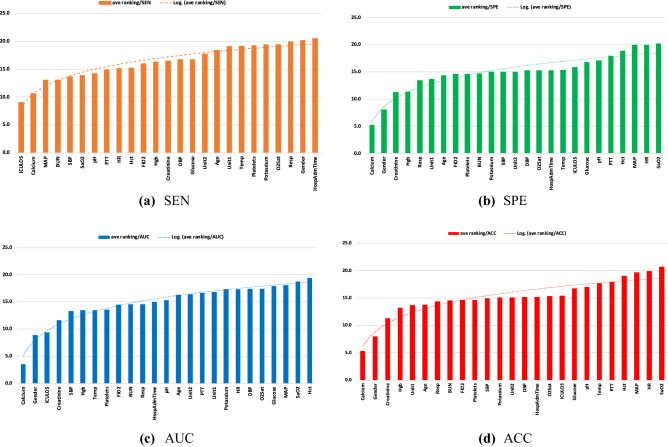
Overall feature importance ranking for BiLSTM predictions under various measures of predictive accuracy.

Finally, we examined the overall importance of features (averaged over all combinations of $h$ and $w$) for predicting sepsis using BiLSTM. Figure 8 illustrates the average importance ranking of the features for each predictive performance measure, where a lower ranking implies higher importance. First, the results suggest that different predictive measures lead to differences in the importance ranking of the features. For instance, while Gender and Creatinine are among the most important predictors for SPE, AUC, and ACC, they are not so for SEN. However, there is agreement on the importance of Calcium among all predictive accuracy measures. Second, the average importance ranking of the features is often larger when the predictive performance measure is SEN, suggesting higher volatility of the importance rankings from the SEN perspectives, particularly among the four most important features. For example, the average ranking of the most important predictors for SEN (i.e., Calcium) is 9, suggesting higher disagreements regarding the importance ranking of Calcium for SEN under various combinations of $h$ and $w$. We conjecture that the difference is relevant to the varying role of $w$ depending on $h$. We observed that while under $w=1$, the importance ranking of Calcium decreases from 1st when $h=1$ to 10th when $h=18$; under $w=6$, the ranking increases from 10th when $h=1$ to 3rd when $h=18$, verifying the hypothesis.

To examine the importance of features, we also used SHapley Additive exPlanation (SHAP) analysis^34^. SHAP is a game theory-based feature analysis that quantifies the independent contribution of each feature to the prediction performance considering the role of other features. The method calculates Shapley values that measure how to fairly distribute the prediction among features^35^. The SHAP feature importance results are presented in Appendix C-VI for $h=1$ and $h=12$ (at $w=1$). The results indicate significant variations of the feature importance between $h=1$ and $h=12$. While the presented SHAP feature importance results pertain to a certain combination of $h$ and $w$, our reported results (Fig. 8) provide a summary of the feature importance over a wider range of $h$ and $w$ based on multiple predictive performance measures, leading to more robust analyses. Moreover, implementing the SHAP feature importance for larger window sizes (i.e., $w$) is computationally extensive.

### Generalization

To assess the inter-cohort predictability, we assessed the predictive performance when different combinations of the two hospitals were considered in the training, while the predictive performance was examined on the hospital with the smaller share. These combinations were 0A + 1B and vice-versa (i.e., the share of smaller hospital = 0%), 0.5A + 0.5B (i.e., the share of smaller hospital = 50%), 0.8A + 0.2B and vice-versa (i.e., the share of smaller hospital = 20%), and 0.9A + 0.1B and vice-versa (i.e., the share of smaller hospital = 10%). The results of average predictive performances over *h* = 1–12 (for *w* = 1) are presented in Appendix C-V. The results indicate that there is a decrease in the predictive accuracy of the minor hospital when the share of the hospital in the learning stage decreases. This has been a consistent observation in most studies using the same data^21^. This is due to the structural differences between the two hospitals, particularly regarding their data collection and data distribution^36,37^. Our investigations indicate that even a small incorporation of the hospitals' data in the learning process (e.g., 10%) provides a better learning opportunity to capture the discrepancies between the two hospitals. This leads to improved diversity of the training data and enhanced predictive performance on the test hospital, hence improved generalizability.

### Impact of data handling

To examine the individual and joint impacts of the two data handling procedures used in this study (i.e., missing data imputation and data resampling), we examined the stage-wise performance of our predictive model under none, either, and both procedures. The results (available in Appendix C-IV) indicate that the contribution of the resampling stage is more than the missing data imputation stage, particularly when emphasizing the importance of predicting the positive class (hence, SEN and AUC).

## Discussion

In this study, we assessed the performance of several ML algorithms in predicting sepsis under various combinations of prediction horizons and observation windows using different predictive performance measures. First, we showed that overall, the BiLSTM algorithm outperforms other ML techniques in predicting sepsis, providing support for the superior performance of this algorithm with complex, longitudinal data^38,39^. Second, using BiLSTM as the benchmark, we found that (i) increasing information recency always boosts the predictive performance; however, the impact of increasing information adequacy depends on the prediction horizon and the purpose of prediction such that (ii) if the prediction is responsive to the positive label (e.g., using SEN and AUC), increasing historical data improves the predictive performance when the prediction horizon is short-moderate, and it reduces the predictive performance when the prediction horizon is long. However, (iii) if the prediction is less responsive to the positive label (e.g., using SPE and ACC), increasing historical data always boosts the predictive performance. Result i supports the importance of data recency to boost the predictive accuracy, which is well-documented in the literature^20,40–45^. According to result ii, increasing the historical information from the oldest part of the window (as we have modeled in our study) acts against information recency. Consequently, enlarging observation windows leads to a tradeoff between the oldness of historical information (negative impact) and its magnitude (positive impact). This result indicates that for SEN and AUC, the benefit of increasing historical information dominates its negative impact up to a certain prediction horizon threshold. After this threshold, the negative impact dominates, leading to reduced predictive performance. Results iii ascertain that there is no such tradeoff when the purpose of prediction is SPE or ACC.

### Comparison of findings with prior literature

A compelling body of previous research has developed models for early sepsis predictions ranging from 1 to 48 h ahead of a sepsis occurence^20,40–43,46–48^. Only a few studies have investigated the effects of the adequacy of historical information (i.e., variation in observation windows) on sepsis prediction^49^. Moreover, limited literature has examined the effects of both information recency and its adequacy on predicting sepsis^44,45^. In one study, Guan et al.^45^ investigated how far ahead sepsis can be detected and how many observations are required to achieve reasonable accuracy. They have assessed the recency and adequacy separately for predicting sepsis using the Sepsis-2 criteria. However, the authors recommended future research to investigate the impact of these two factors when the diagnosis criterion is Sepsis-3, which is more challenging because of the resulting excessive data imbalance. Another study by Scherpf et al. examined the length of the sequence of values (equivalent to $w$ in our study) for different prediction horizons^44^. Guan et al.^45^ concluded that, independently of the prediction horizon, only the last six biometric records are informative for sepsis prediction, and predictive performance remained unchanged when more than six records were included. However, Scherpf et al.^44^ concluded that increasing historical information always boosts predictive performance. Both studies have only used AUC as the predictive measure. Rafie et al.^41^ used the observation window ($w$) intertwined with prediction horizon/window ($h$), masking the impact of the prediction window from that of the observation window on the predictive performance. Our study, however, explicitly segregates these impacts. Based on extensive analytical investigations and using various predictive performance measures, our study extends this scant literature by ascertaining that the impact of information recency and adequacy on predicting sepsis depends not only on the predictive measure of interest (i.e., the purpose of prediction) but also on the intended length of the prediction horizon.

Our study also demonstrated the feasibility of using BiLSTM for the early detection of sepsis. To the best of our knowledge, BiLSTM has not been used previously for sepsis prediction. Although our BiLSTM-based model was designed using a simple architecture with few layers and neurons, it achieved impressive results (average AUC = 0.91, average SEN = 0.80) compared with six other ML algorithms. In the following lines, we describe the studies conducted based on our data (i.e., the open-source sepsis clinical data provided through the PhysioNet challenge 2019). To remain consistent with the sepsis prediction literature, which is predominantly based on the AUC measure (86% of studies according to Fleuren et al.^50^), we excluded the studies, which have not reported AUC. Using this data, Li and colleagues^51^ applied a LightGBM classifier for sepsis prediction and reported an AUC of 0.845, where the sensitivity and specificity were 0.859 and 0.634, respectively. Zabihi and colleagues^52^ developed an ensemble of XGboost models and achieved a relatively similar predictive performance. Yang and colleagues^53^ developed an ensemble of XGBoost models, achieved the AUC value of 0.85 with a higher sensitivity (0.90) and low specificity (0.64). Lee and colleagues^54^ proposed a graph-based convolutional networks, leading to slightly lower predictive performance. Du et al.^55^ applied a gradient boosting tree classifier with a weighted cross-entropy loss function to predict sepsis occurrence within 6 h before its onset. Using a two-stage framework, He et al.^56^ developed an ensemble learning model of LSTM for feature extraction and two methods of gradient boosting machines (XGBoost and gradient boosting decision tree) as the regressor. Lyra et al.^57^ proposed an optimized Random Forest for the prediction of septic patients. Nesaragi and Patidar^58^ employed a ratio and power-based Rusboost model and achieved an AUC of 0.843 on the fivefold cross-validation. The authors extended their works^27,59^ by proposing a unique algorithm for tensor factorization that uses pointwise mutual information, achieving far better predictive performance compared to the previous works (AUC = 0.86). Rafie et al.^41^ proposed an LSTM-CNN model using intertwined prediction and observation windows and achieved the AUROC, specificity, and sensitivity of 0.92, 0.81, and 0.85, respectively, for predicting sepsis four hours before its onset. These results are also comparable to those of Kok et al.^60^ using the temporal convolutional network. Table S1 in Appendix A summarizes the studies discussed above.

Finally, we examined the importance of features for predicting sepsis using BiLSTM based on various measures of predictive performance. In terms of laboratory biometrics, our results agreed with the literature supporting the importance of calcium^61–63^, based on all measures of predictive accuracy, and the level of blood Creatinine^64,65^, based on SPE, AUC, and ACC. However, our results revealed that the importance of features depends on the purpose of the prediction (i.e., the predictive performance criteria).

### Clinical implications

More and more healthcare organizations are deploying predictive analytics to help identify patients with sepsis. When planning the use of these algorithms, clinical teams are often asked to determine a list of patient/disease variables, a “look back” timeline for including those variables in the algorithms, and a “look ahead” timeline for sepsis predictions using those algorithms^45^. The “look back” and “look ahead” windows are related to adequacy and recency, respectively. Hoping for as complete a clinical picture as possible, it is understandable that clinicians may seek to populate the algorithm with as many data points as possible over a long timeframe. However, our study suggests that when the purpose of prediction is being accurate and timely regarding sepsis predictions, if physicians have long prediction horizons, they should not consider the remote information of the patient; instead, they should rely more on the fewer yet more recent clinical data of the patient. Increasing historical information is only useful when the prediction horizon is short-moderate. The goal of predictive analytics in identifying patients with sepsis is ultimately to send notifications to clinicians when patients are at risk. Unacceptable false positives can lead to inappropriate use of medical resources as clinicians are likely to perform follow-up assessments and testing on patients identified as at risk for sepsis. Further, increases in alerts to physicians based on false positives will erode confidence in the algorithm and could result in ignoring future alerts. In hospitals that deploy cognitive computing models for sepsis, the consideration of evaluating the impacts of adequacy and recency could lead to a reduction in false-positive rates and improved clinician confidence in predictive modeling for sepsis.

In addition, the high separability of the estimated risk of sepsis achieved by our top-performing model provides important opportunities for risk-based intervention plans, which assign higher priorities to patients with a higher risk. This will lead not only to better patient outcomes but also better hospital resource planning. This is a crucial step, given that the initiation of interventions (e.g., antibiotic therapy) during the clinical management of sepsis is highly time-sensitive^66^, with every hour of delay leading to decreased survival by 4–8%^67^. It is recommended that early goal-directed therapy be completed within 6 h, which leads to 45% relative risk reduction in mortality rates^67^.

Parsimonious predictive models are especially recommended for real-time clinical applications and critical care decision support systems^68^ and offer numerous advantages. First, such models, when integrated into EMR data, to develop early-onset sepsis risk calculator tools^69^ are simpler to implement clinically in real-time settings and more user-friendly^70^. Therefore, such risk calculators can be conveniently used in bedside settings even with non-EMR data. Second, developing parsimonious models requires less effort for data collection and make better use of non-EMR data for model training when EMR data are not available. The key challenge, however, is the trade-off between models’ predictive performance and their complexity. Sepsis requires critical care; hence, there are more advantages, in terms of real-time applications, associated with models that include input variables, which are routinely collected and are independent of diagnosis time and physician judgement^71,72^. Therefore, examining such a tradeoff in our model and its complexity with fewer yet most informative features is an important avenue in our future research.

## Limitations

First, this was a retrospective cohort study with limited control over data collection. Second, the data used in this study were collected from only two hospitals. Future studies could replicate the prediction and train the models on new data collected from multiple care settings under a controlled environment to generalize the findings. Third, there may have been some other confounders that were not included in our data. Comorbidities are important risk factors for sepsis^67,73^; therefore, the performance of the predictive models might be affected by the history of patient comorbidities. The dataset from the 2019 PhysioNet Computing Challenge did not include other parameters associated with infection, such as c-reactive protein or procalcitonin that are mentioned in the Surviving Sepsis guidelines^74,75^. Further, lactate (lactic acid) remains a feature of the Hour One Surviving Sepsis Bundle but was not included in the model as it had 98% missingness in the dataset. There were several features that had at least 90% missing values that were removed from this modeling. Among these features are PaO2, troponin I, and liver markers. Further, the level of consciousness (LOC) used in the quick SOFA (qSOFA) application of Sepsis-3 was not included. Most sepsis scoring systems delineate between adults (18 years and above) and children. The dataset used for this analysis was made more robust with the inclusion of those under 18 years of age. Given the outcome of results that match other ML-based sepsis predictions, and the relatively low numbers of pediatric patients, we believe that our data is valid. Finally, it is unclear what the definition of gender was within the dataset. It is possible that this refers specifically to biological sex or legal sex and not gender identity.

## Conclusion

In this study, we developed several ML predictive models for predicting sepsis using large longitudinal data under various scenarios of recency and adequacy of historical information. In this way, we demonstrated the superior performance of the DL algorithm—that is, BiLSTM—compared with six examined ML algorithms. Our approach may inform the early identification of patients at the risk of sepsis and provides insights regarding the significance of the recency and adequacy of information for clinical decision-making.

## Supplementary Information

Supplementary Information.

## Data Availability

The dataset used for analyses in the current study has been published and made publicly available^21^. We have also made the dataset available on the GitHub repository designed for this study (https://github.com/bi-Lstm-for-sepsis-prediction/bi-Lstm-for-sepsis-prediction.git). The password for accessing these items is *Sepsis2021*.
