# Supplementary material for: The impact of recency and adequacy of historical information on sepsis predictions using machine learning
Source: Sci Rep. 2021 Oct 21;11:20869. doi: 10.1038/s41598-021-00220-x (PMC8531301; doi:10.1038/s41598-021-00220-x)
Supplement: Supplementary file 1 — Supplementary Information. [file 41598_2021_220_MOESM1_ESM.pdf]

# The Impact of Recency and Adequacy of Historical Information on Sepsis Predictions Using Machine Learning

**CONTENTS:**


**APPENDIX A: LITERATURE SUMMARY** ........ **2**

**APPENDIX B: DESCRIPTIVE RESULTS** ........ **3**

**APPENDIX C: TECHNICAL RESULTS** ........ **4**

I. Details of Hyperparameter Tuning ........ 4

II. Details of Fβ Performance Metric........ 4

III. Pseudocode for modified MICE ........ 5

IV. Details on the Impact of Data Handling Procedures........ 6

V. Details on Generalization Analysis........ 6

VI. Details on the SHAP Feature Importance........ 7

VII. Details on the Features' Missingness........ 8

**REFERENCES**........ **9**

## APPENDIX A: LITERATURE SUMMARY

**Table S1** Summary of studies conducted on the same data (PhysioNet Challenge 2019)

| Authors, Year | Method | Performance measures |
|---|---|---|
| Li et al., 2020 [1] | Time-phased Model on LightGBM | AUC: 0.845<br>Sensitivity: 0.859<br>Specificity: 0.634 |
| Zabihi et al., 2019 [2] | Ensemble of XGboost models | AUC: A (0.814); B (0.844)<br>AUPRC: A (0.102); B (0.110)<br>F1-measure: A (0.128); B (0.130) |
| Yang et al., 2020 [3] | Ensemble of XGBoost models | AUC: 0.85<br>Sensitivity: 0.90<br>Specificity: 0.64 |
| Lee et al., 2020 [4] | Graph-based convolutional networks | AUC: A (0.802); B (0.815)<br>AUPRC: A (0.097); B (0.090)<br>F1-measure: A (0.156); B (0.139) |
| Du et al., 2019 [5] | Gradient boosting tree + weighted cross-entropy loss function | AUC: A (0.811); B (0.853)<br>AUPRC: A (0.105); B (0.119)<br>F1-measure: A (0.131); B (0.142) |
| He et al., 2020 [6] | Ensemble model of LSTM, XGBoost, GBDT | Sensitivity: 0.640<br>Specificity: 0.844<br>AUC: A (0.816); B (0.847) |
| Lyra et al., 2019 [7] | Random Forest | AUC: A (0.788); B (0.828)<br>AUPRC: A (0.083); B (0.089)<br>F1-measure: A (0.121); B (0.132) |
| Nesaragi & Patidar, 2020[8] | Ratio and Power-based Rusboost | AUC: 0.843 |
| Nesaragi et al., 2021[9] | LightGBM+ Tensor factorization | AUC=0.8621<br>F1-measure =0.1638 |
| Nesaragi et al., 2021[10] | | AUC=0.8613<br>F1-measure =0.1307 |
| Rafie et al., 2121 [11] | LSTM-CNN model | AUC: 0.92<br>Specificity: 0.81<br>Sensitivity: 0.85 |
| Kok et al., 2020[12] | Temporal Convolution Network | AUC=0.91 |

**Notes:** GBDT= gradient boosting decision tree, LSTM: Long short-term memory, XGBoost: extreme gradient boosting, RNN: recurrent neural network, A: Hospital A, B: Hospital B

## APPENDIX B: DESCRIPTIVE RESULTS

**Table S1:** Descriptive results

| Variables | Mean (SD) or Frequency (%) | |
|---|---|---|
| | **Sepsis (n=27,916)** | **Non-Sepsis (n=1,524,294)** |
| **Vital signs** | | |
| Heart rate (beats per minute) | 195.8 (126.1) | 216.8 (113.8) |
| Pulse oximetry (%) | 93.4 (59.3) | 92.3 (59.3) |
| Temperature (Deg C) | 158.7 (169.3) | 123.2 (149.6) |
| Systolic BP (mm Hg) | 256.6 (244.4) | 225.4 (220) |
| Mean arterial pressure (mm Hg) | 528.8 (286.2) | 477.8 (294.5) |
| Diastolic BP (mm Hg) | 303.5 (121.6) | 314.8 (142.2) |
| Respiration rate (breaths per minute) | 40.1 (30.3) | 31.7 (26.4) |
| **Laboratory values** | | |
| Fraction of inspired oxygen (%) | 17.6 (24.5) | 8.3 (16.8) |
| pH | 17.7 (27.5) | 8.6 (20.4) |
| Oxygen saturation from arterial blood (%) | 30.9 (80.0) | 11.8 (46.5) |
| Blood urea nitrogen (mg/dL) | 24.7 (52.8) | 14.3 (40.6) |
| Calcium (mg/dL) | 46.3 (125.9) | 32 (110.9) |
| Creatinine (mg/dL) | 26.4 (119.1) | 12.7 (77) |
| Serum glucose (mg/dL) | 105.3 (236.8) | 80.4 (214.6) |
| Potassium (mmol/L) | 28.1 (57.5) | 16.3 (43.3) |
| Hematocrit (%) | 68.5 (129.3) | 46.9 (112.9) |
| Hemoglobin (g/dL) | 32.0 (77.9) | 19.1 (60.3) |
| Partial thromboplastin time (seconds) | 61.1 (187.8) | 32.7 (137.1) |
| Platelets (count*10^3/µL) | 50.7 (147.3) | 28.3 (105.1) |
| **Demographics** | | |
| Age | 62 (16.4) | 62 (16.3) |
| Sex † | | |
| Male | 16,563 (59.3%) | 851,540 (55.9%) |
| **Admin Variables** | | |
| Admin. identifier for Medical ICU† | | |
| Yes | 9,127 (32.7%) | 457,774 (30.0%) |
| Admin. identifier for Surgical ICU† | | |
| Yes | 6,206 (22.2%) | 467,143 (30.1%) |
| Hours between hospital admit and ICU admit | 1013.4 (1946) | 983.6 (1951) |
| ICU length-of-stay (hours since ICU admit) | 55.7 (59.2) | 26.5 (27.9) |
| † Categorical variable | | |

# APPENDIX C: TECHNICAL RESULTS

## I. Details of Hyperparameter Tuning

**Table S2** Details of hyperparameter space, optimization approach, and optimal values for BiLSTM

| | Hyperparameter Configuration | |
|---|---|---|
| **Parameters** | **Ranges** | **Optimal Value/Choice** |
| Activation functions | ELU, ReLU, LeakyReLU, Tanh | ELU |
| Learning rate | [1e-1, 1e-5] | 1E-02 |
| Optimizers | SGD, RMSprop, Adam, Adagrad | Adam |
| Epochs | [10-300] | 50 |
| Neuron | [10-600] | 130 |
| Drop-out | [0.1-0.8] | 0.5 |
| Merge mode | Average, Concat | Concat |
| Batch size | 100-5000 | 1024 |
| Early Stopping | [1-10] | 4 |

## II. Details of Fβ Performance Metric

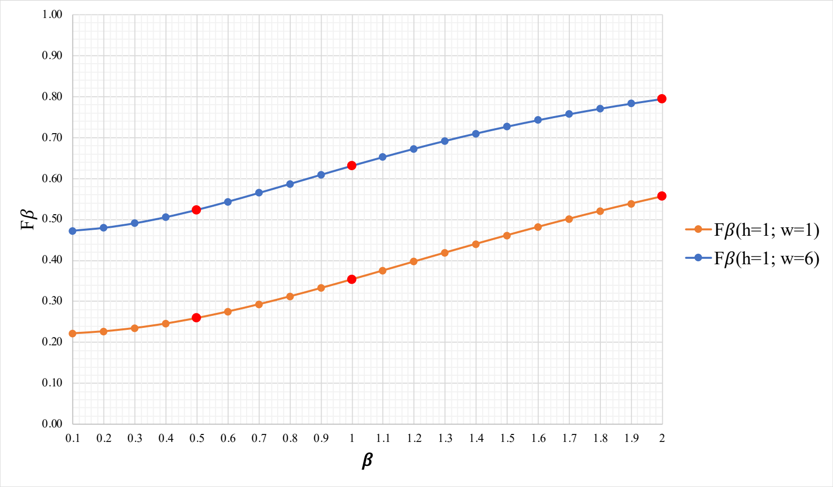


**Figure 1-** Fβ for h=1

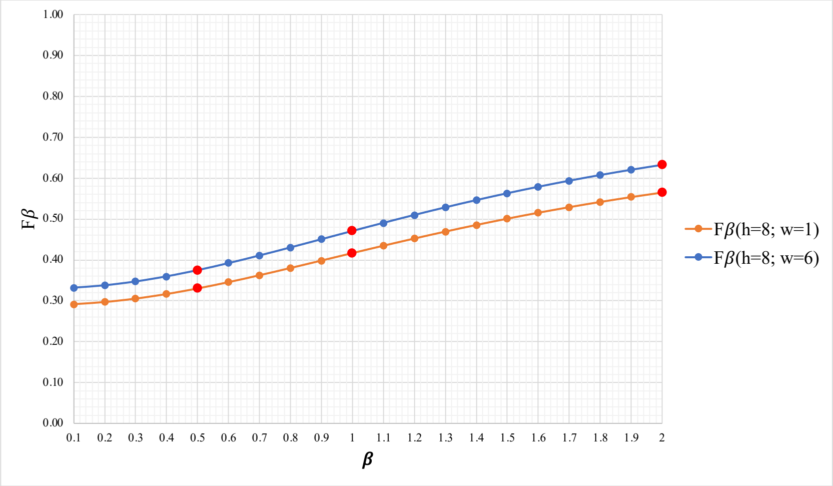


**Figure 2-** Fβ for h=8

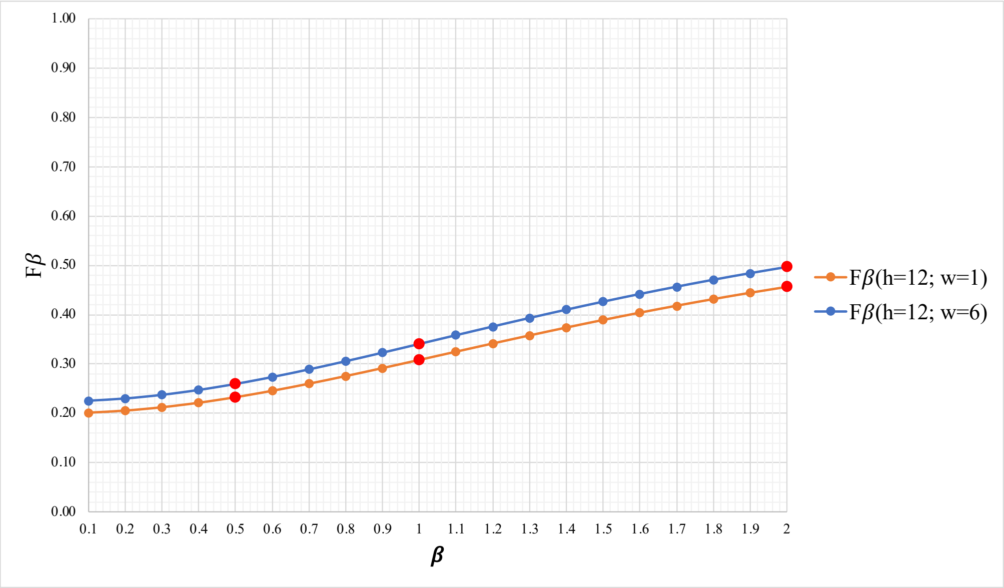


**Figure 3-** Fβ for h=12

## III. Pseudocode for modified MICE

---

**Forward MICE Algorithm: Applying MICE Imputation on Time Series without Using Future Data**

---

**1. Input:** Dataset (size = records×features)

**2. Output:** Imputed Dataset (size = records×features)

<u># Parameters:</u>

**3. WINDOWSIZE** = 4 *# this is B in the manuscript, it could be set to any other value in the expense of complexity*

**4. initial_data** = **Input** [feature name: 'ICULOS'] = = (1, WINDOWSIZE) *# select rows, where ICULOS = 1–WINDOWSIZE*

**5. imputed_dataset** = **MICE** (**initial_data**)

**6. min** = 1

**7. max_ICU** = **200**

<u># loop</u>

**8. For** step **in** range (**min**, **max_ICU**) **do**

**9.** **M** = **Input** [feature name: 'ICULOS'] == (step, step + WINDOWSIZE) *# select rows, where ICULOS = step – (step + WINDOWSIZE)*

**10.** **N = MICE(M)**

**11.** **O = N** [feature name: 'ICULOS'] == (step + WINDOWSIZE) *# select rows, where ICULOS =(step + WINDOWSIZE)*

**12.** Add **O** to **imputed _dataset**

---

# IV. Details on the Impact of Data Handling Procedures

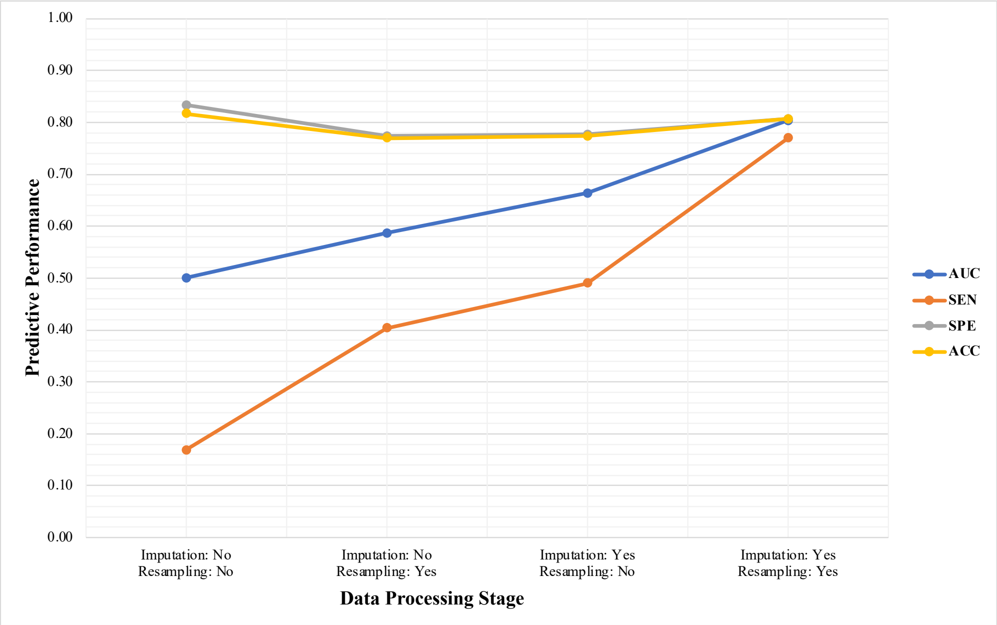


**Figure 4-** The impact of missing data imputation and resampling procedures on model performance averaged over h=1–12 (w=1)

# V. Details on Generalization Analysis

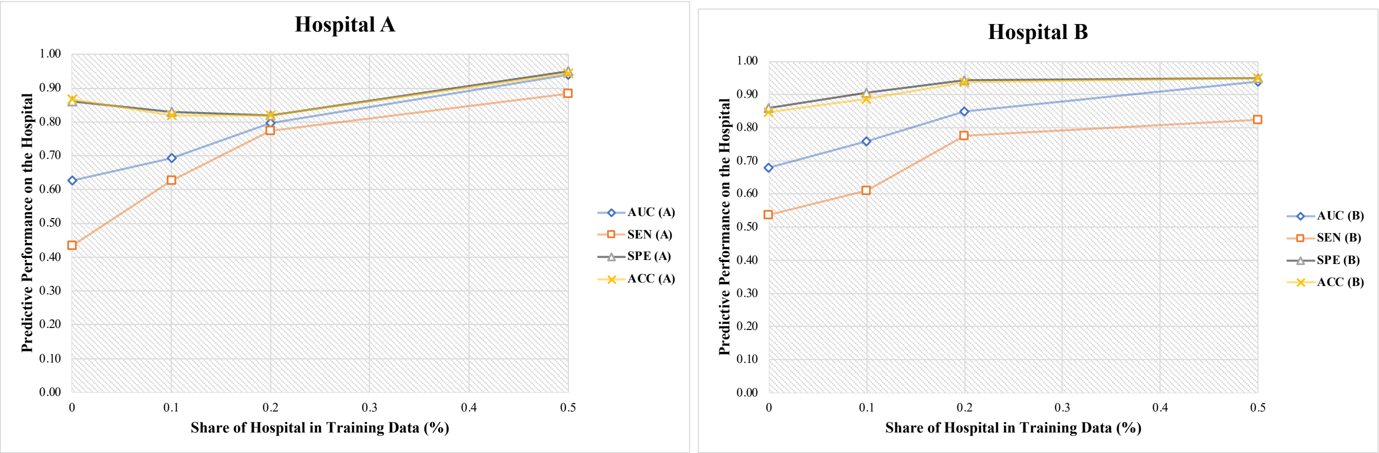


**Figure 5-** Performance measures for multiple data incorporation strategies overaged over h=1–12 (w=1)

# VI. Details on the SHAP Feature Importance

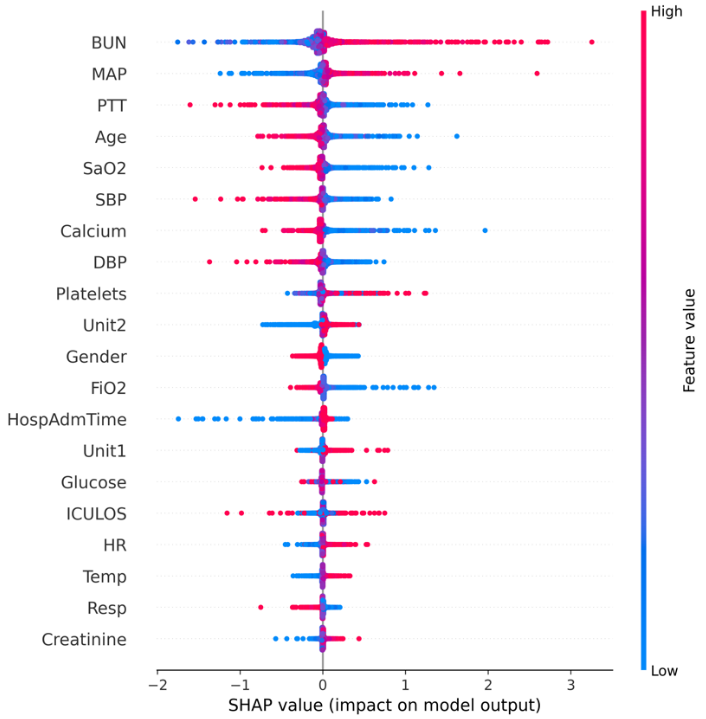


**Figure 6-** Feature importance for h=1 (w=1)

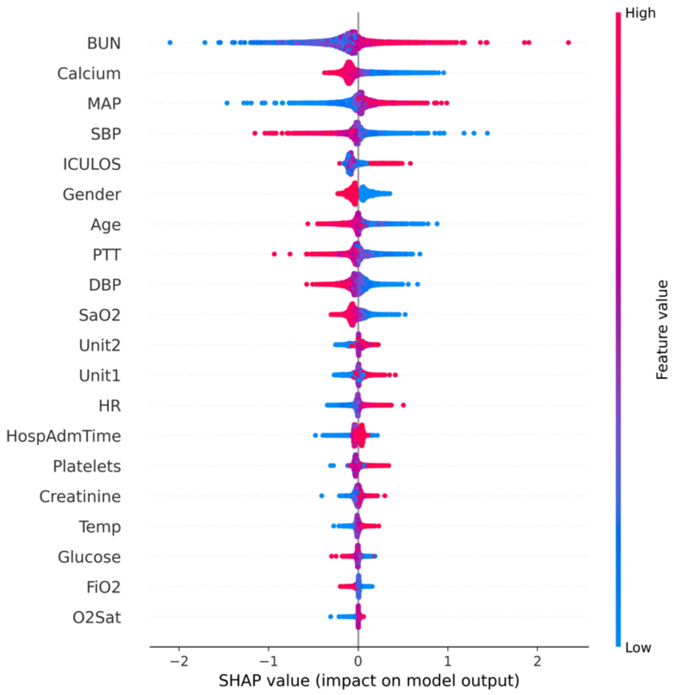


**Figure 7-** Feature importance for h=12 (w=1)

# VII. Details on the Features’ Missingness

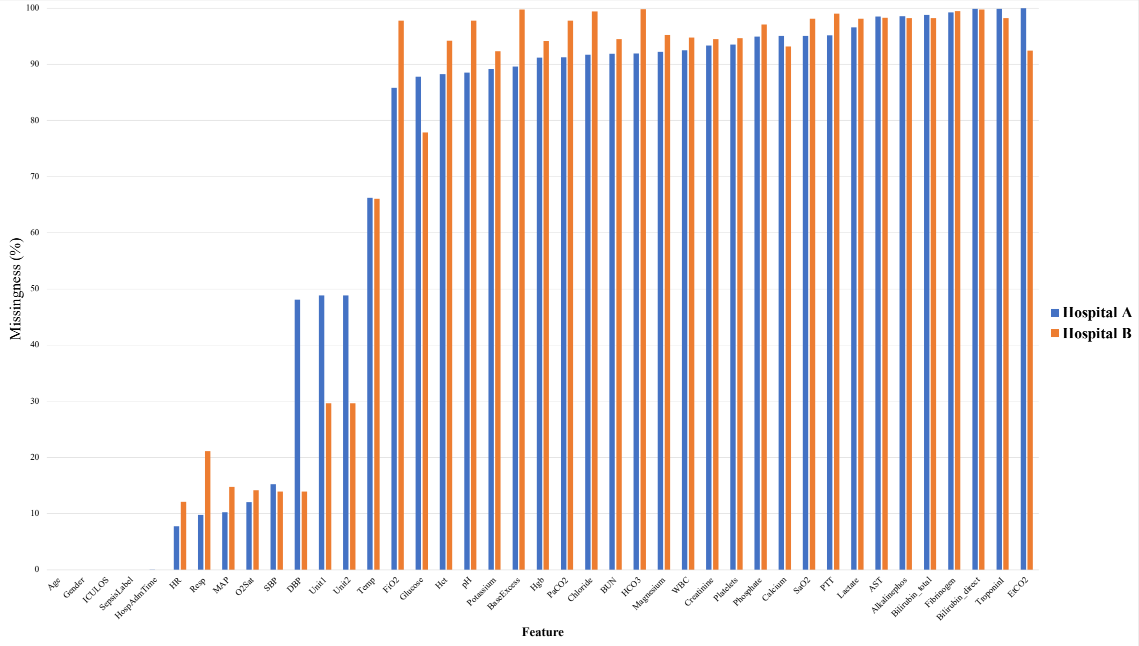


**Figure 8-** The distribution of features’ missingness

## REFERENCES


1. Li, X. *et al.* A time-phased machine learning model for real-time prediction of sepsis in critical care. *Critical Care Medicine* **48**, e884–e888 (2020).
2. Zabihi, M., Kiranyaz, S. & Gabbouj, M. Sepsis prediction in intensive care unit using ensemble of XGboost models. in *2019 Computing in Cardiology (CinC)* Page-1 (IEEE, 2019).
3. Yang, M. *et al.* An explainable artificial intelligence predictor for early detection of sepsis. *Critical Care Medicine* **48**, e1091–e1096 (2020).
4. Lee, B. T. *et al.* Graph convolutional networks-based noisy data imputation in electronic health record. *Critical Care Medicine* **48**, e1106–e1111 (2020).
5. Du, J. A., Sadr, N. & de Chazal, P. Automated prediction of sepsis onset using gradient boosted decision trees. in *2019 Computing in Cardiology (CinC)* Page-1 (IEEE, 2019).
6. He, Z. *et al.* Early sepsis prediction using ensemble learning with deep features and artificial features extracted from clinical electronic health records. *Critical Care Medicine* **48**, e1337–e1342 (2020).
7. Lyra, S., Leonhardt, S. & Antink, C. H. Early prediction of sepsis using random forest classification for imbalanced clinical data. in *2019 Computing in Cardiology (CinC)* 1–4 (IEEE, 2019).
8. Nesaragi, N. & Patidar, S. Early prediction of sepsis from clinical data using ratio and power-based features. *Critical Care Medicine* **48**, e1343–e1349 (2020).
9. Nesaragi, N., Patidar, S. & Thangaraj, V. A correlation matrix-based tensor decomposition method for early prediction of sepsis from clinical data. *Biocybernetics and Biomedical Engineering* **41**, 1013–1024 (2021).
10. Nesaragi, N., Patidar, S. & Aggarwal, V. Tensor learning of pointwise mutual information from EHR data for early prediction of sepsis. *Computers in biology and medicine* **134**, 104430 (2021).
11. Rafiei, A., Rezaee, A., Hajati, F., Gheisari, S. & Golzan, M. SSP: Early prediction of sepsis using fully connected LSTM-CNN model. *Computers in biology and medicine* **128**, 104110 (2021).
12. Kok, C. *et al.* Automated prediction of sepsis using temporal convolutional network. *Computers in Biology and Medicine* **127**, 103957 (2020).
